# 

**Published:** 1888-09

**Authors:** 


ANNOUNCEMENT.

After this issue the office of the INDEPENDENT PRACTITIONER will be removed from Buffalo to 1215 Filbert St., Philadelphia, to which address all editorial and business correspondence directly pertaining to the management of the journal may be sent. The office of the Secretary of the Association, Dr. Geo. S. Allan, is 51 West 37th St., N. Y. city, and any business correspondence relating to the Association may be sent to him. The journal is cosmopolitan in character. The Association is chartered in New Jersey, with the secretary's office in New York, while the editorial work is done in Philadelphia. It counts among its stockholders an equal number of Philadelphia and New York men, while shares are scattered largely in the New England, middle and western States and foreign countries. No change will be made in the dress or make-up of the journal until the beginning of Vol. X, January 1, 1889. The name will then be changed to THE INTERNATIONAL DENTAL JOURNAL. We hope by that time to have our list of foreign contributors completed, as Dr. W. D. Miller has very kindly undertaken to organize them. All our stockholders will naturally give their productions to us, and a glance at the list is all that is needed to guarantee the character of the future contributions of the journal. The number of writers in the dental profession are very limited, especially those who are doing original work, and we feel safe in saying that the PRACTITIONER will hold its own as a strictly independent scientific journal in the future as it has in the past.

The offer of Dr. Stowell's Atlas in connection with renewals or new subscriptions still holds. If you have one already and are satisfied with it, which you cannot otherwise than be, please tell some brother practitioner. Don't keep all your good things to yourself, but share them with your neighbors.

We will begin a full report of the union meeting of the Massachusetts and Connecticut Valley Societies, held at Boston, July 10, 11, 12 and 13, 1888, in the October number. Those who desire extra copies should send in their order soon.

---

SPECIAL ANNOUNCEMENT.

According to the terms of the transfer of the INDEPENDENT PRACTITIONER to the new syndicate, all subscriptions due the old syndicate for the current year are to be paid to the old syndicate.

If you are in arrears on your subscription which ran out July, 1888, or will close January, 1889, please send the amount of your indebtedness directly to Dr. Barrett, as we have nothing whatever to do with the business of the New York Dental Journal Association. All the bills and letters that you may have received have been sent by them and not by us. We are led to make this statement because parties have written to us enclosing money which did not belong to us. Of course, if we can do anything to accommodate our subscribers, we shall be glad to do so, but it will be better for you to send money due the New York Dental Journal Association directly to Dr. Barrett, as he has the books, and is conversant with the accounts, and, at best, we could only send the money on to him.

### The Independent Practitioner—Vol. IX.

### PUBLISHED BY DENTISTS FOR DENTISTS.

### Prospectus.

With the present number THE INDEPENDENT PRACTITIONER assumes a more important role, as the organ of the International Dental Journal Association. The promises of the New York Dental Journal Association of making volume IX better than any former volumes, have been well carried out. The scope of the Journal has been enlarged, and its circle of influence greatly increased. We still continue the magnificent offer of Dr. Stowell's work on the *Microscopic Structure of a Human Tooth*, which is published at the moderate price of six dollars each. We have made such arrangements with the publishers that we can furnish it in connection with a subscription to **The Independent Practitioner for One Dollar and Fifty Cents. That is, to every one who will send in Four Dollars we will send the Independent Practitioner one year and a copy of this beautiful and useful atlas.**

This offer is intended only for subscribers in America. Foreign residents within the postal union must enclose fifty cents extra for prepayment of postage upon the Journal. Our regular rate of subscription to such is three dollars per annum.

As the book absolutely costs more than the sum for which it will be furnished, the money must accompany the subscription. Nor will the book be sold separately. It will only be sent in connection with a subscription to this journal.

The book will be sent either by mail or express. If it is desired that it should be sent by mail, the postage (twenty-five cents) should be sent. All remittances should be sent to the Editor and Business Manager, Dr. W. X. Sudduth, 1215 Filbert Street, Philadelphia, Pa. Postal Orders, Postal Notes, or New York Drafts are the safest and most convenient.

THE INDEPENDENT PRACTITIONER has been transferred to a new and enlarged syndicate—no material change is, however, to be made in the form of the journal until the close of the present volume. We hope to hold all our old subscribers, and obtain many new ones. You may hand your subscription to any one of the stockholders, a list of whose names is here appended.

GEO. S. ALLAN
R. R. ANDREWS
H. A. BAKER
W. C. BARRETT
A. P. BEALE
S. T. BEALE
C. S. BECK
G. V. BLACK
E. A. BOGUE
C. F. W. BODECKER
W. G. A. BONWILL
EDWARD C. BRIGGS
THOS. BRADLEY
ALBERT H. BROCKWAY
WM. CARR
DWIGHT M. CLAPP
JOHN D. CODMAN
CHAS. D. COOK
EDW. T. DARBY
D. M. DAVIDSON
WM. H. DWINELLE
CHAS. J. ESSIG
J. N. FARRAR
T. FILLEBROWN
W. B. FINNEY
T. A. FLETCHER
M. W. FOSTER
CHAS. E. FRANCIS
W. F. FUNDENBERG
W. H. FUNDENBERG
E. C. FUNDENBERG
E. S. GAYLORD
J. P. GERAN
S. H. GUILFORD
JOHN J. HART
O. E. HILL
J. F. P. HODSON
J. MORGAN HOWE
ROBERT HUEY
LOUIS JACK
G. L. S. JAMESON
C. A. KINGSBURY
EDW. C. KIRK
W. T. LA ROCHE
W. K. LEECH
WILBUR F. LITCH
J. BOND LITTIG
BENJAMIN LORD
GEO. W. LOVEJOY (Canada)
JAMES McMANUS
E. V. McLEOD
DAN'L NEAL McQUILLAN
HORATIO C. MERIAM
W. D. MILLER (Berlin)
W. E. PAGE
S. B. PALMER
C. B. PARKER
S. G. PERRY
C. N. PEIRCE
CHAS. E. PIKE
W. A. PHREANER
W. E. PINKHAM
H. C. REGISTER
L. D. SHEPARD
EUGENE H. SMITH
B. HOLLY SMITH
S. G. STEVENS
W. X. SUDDUTH
AMBLER TEES
J. D. THOMAS
JAMES TRUMAN
WM. H. TRUEMAN
F. F. VAN WOERT
W. W. WALKER
I. T. WARDWELL
C. S. WARDWELL
G. W. WARREN
L. S. WATERS
GEO. W. WELD
JULIUS G. W. WERNER
JACOB L. WILLIAMS
R. B. WINDER
C. A. WOODWARD
J. A. WOODWARD

